# Effect of nutrients and salinity pulses on biomass and growth of *Vallisneria americana* in lower St Johns River, FL, USA

**DOI:** 10.1098/rsos.140053

**Published:** 2015-02-11

**Authors:** Ronald G. Boustany, Thomas C. Michot, Rebecca F. Moss

**Affiliations:** 1 US Geological Survey, National Wetlands Research Center, 700 Cajundome Boulevard, Lafayette, LA 70506, USA; 2 Five Rivers Services, LLC, US Geological Survey, National Wetlands Research Center, 700 Cajundome Boulevard, Lafayette, LA 70506, USA

**Keywords:** submerged aquatic vegetation, nutrients, salinity, *Vallisneria americana*, St Johns River

## Abstract

We determined the interactive effects of nutrient loading and salinity pulsing on *Vallisneria americana* Michx., the dominant submerged aquatic vegetation species in the lower St Johns River (LSJR), FL, USA, and its associated algal community. Five hundred and ninety 6-inch diameter intact plant plugs of *Vallisneria* were collected from the LSJR in March 2003 and transported to US Geological Survey mesocosm facilities in Lafayette, LA, USA. A 3×3 experimental design consisting of three nutrient levels (control, 1/3 control and 3× control) and three salinity pulsing regimes (no pulse, 1-pulse at 18 ppt and 2-pulse at 12 and 18 ppt) was implemented with three replicates per treatment for a total of 27 experimental tanks. Salinity pulsing significantly reduced all measured *Vallisneria* growth parameters including above- and below-ground biomass, areal productivity and leaf area index. Nutrient levels had little effect on plants subjected to salinity pulses, but in non-salinity pulse treatments we observed higher mean macrophyte biomass in the low-nutrient loading treatments. Macroalgal components (epiphytes and surface algal mats) were not significantly different ( *p*=0.2998 and *p*=0.2444, respectively), but water column chlorophyll *a* (phytoplankton) was significantly higher ( *p*<0.0001) in all salinity pulse treatments except for the 1-pulse, low-nutrient treatment. A single salinity pulse at 18 ppt resulted in 22% pot mortality and two consecutive pulses of 18 and 12 ppt resulted in an additional 14% mortality. Individual leaves and ramets lost 59.7% and 67.8%, respectively, in the combined salinity pulse treatments. Nutrient loading tends to have a long-term effect on *Vallisneria* through complex community interactions while salinity pulsing frequency and intensity has an immediate and direct influence on growth and distribution.

## 2. Introduction

The St Johns River is one of the largest rivers in the state of Florida, encompassing a 9562 square mile drainage area. The lower St Johns River (LSJR) basin extends slightly more than 100 miles and can be differentiated into three riverine, salinity and limnologic zones: fresh tidal lacustrine, predominantly oligohaline lacustrine and mesohaline riverine. The southern (upstream) portion of the basin is largely rural and land is used primarily for forestry, row crop production and nurseries, while the northern portion of the basin is heavily urbanized, containing the municipalities of Jacksonville, Orange Park and Middleburg [1].

Although land use is variable throughout the basin, the entire length of the river has been adversely impacted in some form or another. Since the late 1950s, a series of water quality problems related to point and non-point source pollution have been identified and addressed within the LSJR basin. Various enacted initiatives were effective at the time; however, the continual rapid expansion of development and regional population growth has raised concerns regarding potential degradation of water quality. These concerns have led to recent water quality management initiatives by the St Johns River Water Management District (District) to set basin-wide thresholds of pollution loading in order to restore or maintain aquatic ecosystem health in a cost-effective and equitable manner [1]. These District initiatives are designed to protect the natural resources of the river and to maintain a high quality and productive ecosystem for the benefit of the surrounding communities.

Paramount to the integrity of the ecosystem is sustaining the submerged aquatic vegetation (SAV) community that supports the abundant microfauna and fisheries of the river. SAV is considered to be one of the most important biological components of the LSJR [2]. The dominant SAV species in the LSJR, *Vallisneria americana* Michx., constitutes approximately 62% of the SAV coverage in the basin [3]. Basin-wide anthropogenic activities that include non-point source nutrient loading and hydrologic alterations have induced environmental changes that currently threaten this valuable resource. Therefore, an understanding of factors influencing the distribution and health of *Vallisneria* is fundamental to developing sound management practices for the restoration and protection of this important resource [4].

Salinity is one of the most prevalent factors determining SAV distribution in estuarine ecosystems. The LSJR is essentially estuarine, but the extent of salinity influence varies dramatically. In fact, the bed of the river remains below sea level for 400 of 500 km length, which makes the lower basin subject to tidal activity and occasional reverse flow events resulting from low discharge rates during droughts or winds associated with persistent weather fronts [5]. Saline water can intrude as much as half the length of the river upstream.

Unlike most other environmental factors, the effects of salinity on freshwater species are profound, and thus delineation of strictly freshwater species is indicative of the farthest reaches of saline water. Although *Vallisneria americana* Michx. is considered a freshwater species [6–8], it has been documented on numerous occasions to tolerate moderate levels of salinity [9–19]. Yet there remains some uncertainty regarding the salinity tolerance limits of *Vallisneria*. Salinity tolerance limits have been reported in the range from 8 to 20 ppt providing evidence that tolerance limits may vary from population to population [14,20]. Differing methodologies may partially explain the variations in salinity tolerance, in particular the intensity, frequency and duration of exposure, but evidence suggests that populations may differ and predicting the tolerance of a given *Vallisneria* population to salinity may require study of individual populations and circumstances.

Other factors may influence the ability of *Vallisneria* to tolerate episodic salinity exposure. The adverse effect of excessive nutrients on SAV communities has been well documented [21–24], including the increase in competitive algal components and the subsequent reduction in critical light required for SAV growth. A less understood aspect to increased nutrient loading on SAV communities is the combined effect that nutrients may have on the ability of the macrophytes to tolerate episodic perturbations, such as salinity pulses. In this study, we evaluated the growth response of *Vallisneria* to three different salinity pulse regimes and three nutrient loading rates by determining (i) the effect of salinity pulses at variable frequency and magnitude, (ii) the effect of nutrients on *Vallisneria* salinity pulse tolerance and (iii) the interactive effects of salinity pulses and nutrients on *Vallisneria* and associated algal components.

## 3. Material and methods

### 3.1 Collection

Approximately 590 *Vallisneria* plant plugs were collected from the St Johns River at Eagle Point on 25 March 2003. The intact plant plugs were extracted with 6-inch (15-cm) diameter coring tubes and transferred immediately to 15 cm diameter pots. The pots were placed in 10-gallon Rubbermaid tubs (six pots per tub) and covered with wet cloths and lids to prevent plant desiccation. The tubs were then transported by boat to the boat launch, loaded into an enclosed trailer and transported to greenhouse facilities at the National Wetlands Research Center (NWRC) in Lafayette, Louisiana. The collection process was completed in 2 days. Upon arrival at NWRC, plants were immediately placed in 120-cm diameter fibreglass tanks with 1 ppt salinity water adjusted to a depth of 76 cm. Twenty pots were randomly placed in a cluster at the centre of each tank. The leaves of each pot were then clipped to approximately 15 cm above the sediment line to improve initial equitability in plant biomass.

### 3.2 Experimental design

The experimental design consisted of nine treatments with three treatment levels for each nutrient loading and salinity pulsing and three replicates for each treatment. Experimental nutrient loading levels were based upon loading rates developed by SJR Water Management District (District) model simulations of seasonal inorganic nutrient consumption patterns [1]. The selected range of salinity pulsing, as specified by the District, was considered the range of concern in light of increased freshwater use in the upstream with rapid commercial and residential development coupled with periods of extreme drought. 18 ppt was considered the upper limit of concern. The three nutrient treatments consisted of a control level, low (1/3 control) and high loading rates (3× control) (table 1). The salinity pulse treatments included (i) control (0-pulse) treatment, in which the salinity was held at 1 ppt throughout the experiment, (ii) 1-pulse treatment at 18 ppt and (iii) 2-pulse treatments at 18 ppt and 12 ppt. We used 12 ppt on the second pulse to differentiate the 2-pulse treatments from the initial 18 ppt 1-pulse treatment. The resulting experimental design (3 nutrient loading rates × 3 salinity pulse levels × 3 replicates) consisted of a total of 27 tanks.

**Table 1.** Experimental nutrient loading rates. All values in μg l^−1^ d^−1^.

nutrient	control	low all	high all
NH_4_	10.75	3.19	32.25
NO_3_	20.3	6.77	60.9
PO_4_	8.43	2.81	25.29

Control conditions consisting of a stock solution of tap water mixed with Forty Fathoms Crystal Sea Marine Mix to a salinity of 1 ppt and nutrients at a concentration equivalent to field conditions was used in all experimental tanks. Field nutrient concentrations were provided by the District. Two years of river monitoring data from 1997 to 1998 were used to determine a mean concentration for ammonium (0.026 mg l^−1^), nitrate (0.129 mg l^−1^) and phosphate (0.041 mg l^−1^). Nutrients were added to each tank initially at the level of these mean concentrations and during acclimation the control nutrient loading rate was applied to all tanks (table 1). Upon experimental initiation, the specified nutrient experimental loading rates were then applied.

Throughout the experiment, water within each experimental tank was circulated continuously using submersible pumps. Chiller units (Aquanetics AFC-3B) were used to regulate the water temperature in the experimental tanks to maintain temperatures corresponding to seasonal trends on the LSJR as recorded by the SJR Water Management District. One-fifth of the volume of each tank was removed biweekly and replaced with the control solution to mimic water exchange. The sides of the tanks were scrubbed weekly to prevent development of algal growth on these artificial substrates.

### 3.3 Sampling and analysis procedures

Within the first week, leaves and ramets (whole plants) of *Vallisneria* were counted in each pot to determine initial conditions. Plants were acclimated to greenhouse conditions for three weeks at control nutrient levels and salinity. Following this three-week acclimation period, ‘pre-treatment’ plants from each tank were randomly selected and harvested. Following the pre-treatment harvest, the nutrient and first salinity pulse treatments were initiated. The second salinity pulse was initiated 2.5 months after experiment initiation and the final harvest took place 4.5 months after experiment initiation. Monitoring indicated that the mean peak salinity in the first pulse was 18.5±0.3 ppt and the second pulse was 11.6±0.2 ppt. Each pulse duration was approximately two weeks, where salinity was increased to treatment level within 3 days, maintained for 2 days and then reduced steadily over a period of two weeks to approximately 1 ppt. The first pulse treatment salinity was reduced at a rate of approximately 1.45 ppt per day over a period of 10 days and the second pulse treatment was reduced at the same rate for the first 4 days then reduced to 0.5 ppt per day over the course of the following 7 days. Because we removed the same volume of water each time to reduce the salinity following salinity pulse treatments, the reduction in salinity was proportional to the concentration. Therefore, salinities dropped at a faster rate initially and tapered off towards the end of the two-week drop period.

During each harvest, four pots were randomly selected from each tank for measurements of above- and below-ground biomass (grams dry weight per metre square (gdw m^−2^) and root : shoot ratio (below-ground biomass divided by above-ground biomass). The sediment was washed from the roots and the above- and below-ground biomass was separated, dried at 60°C for at least one week, and weighed. Areal productivity (grams dry weight of new leaf growth per metre square per day (gdw m^−2^ d^−1^) was determined by randomly selecting two pots from each tank and notching all leaves 2 cm above the sediment line. The pots were replaced in the tanks, and after a period of approximately two weeks, all leaves were harvested and separated into old and new growth, dried and weighed. All new growth occurring below the 2 cm notched area on existing leaves and all newly sprouted leaves were considered new vegetation.

Epiphyte biomass and leaf area index (LAI) were determined from plants in two additional, randomly selected pots using a methodology similar to that described by Neckles *et al.* [25]. All above-ground material was carefully removed and placed into sealed plastic bags for transport to the laboratory. In the laboratory, the leaves were individually scraped in a pyrex dish using a glass slide to remove epiphytic material and then encased in a sheet of polyethylene (Glad Cling Wrap) for leaf area determination. The plastic wrap served as a clear mount for numerous leaves, which could then be passed collectively through a Licor (Model LI3000A) leaf area meter without damage. The LAI (area of leaf/area of pot) was determined by dividing the leaf area by the area of the pots. The scraped epiphyte material was then placed into aluminium pans, dried and weighed as grams dry weight of epiphytes per square centimetre of leaf area (gdw cm^−2^). Leaf and whole-plant counts (ramets) were also recorded for each pot harvested.

Weekly photosynthetically active radiation (PAR) measurements were taken at the surface and at 37 cm depth. A method by Wetzel & Likens [26] was used to determine irradiance where solar irradiance *I*_z_ at depth *z* is a function of the intensity at the surface, *I*_o_, multiplied by the antilog of the negative extinction coefficient (*η* or *K*_d_) at depth *z* in metres

$$\begin{array}{rl} I_{z} & =I_{o}e^{-\eta z}\;\text{or}\\ \ln I_{o}-\ln I_{z} & =\eta z\\ \eta & =\frac{\ln I_{o}-\ln I_{z}}{z}. \end{array}$$

Weekly dissolved oxygen, temperature, pH and salinity measurements were taken with a YSI 650 MDS hand-held meter equipped with a 600 QS multi-parameter water quality probe. Chlorophyll *a* was determined by a modified procedure from Wetzel & Likens [26]. A known volume of water was filtered through a membrane filter (25 mm diameter, 0.45 μm pore size) and chlorophyll *a* was then extracted from the filter with 90% acetone. Fluorescence was measured on a Turner Designs Fluorometer 10-AU [27].

### 3.4 Statistical analysis

Data were analysed (*α*=0.05) using analysis of variance (ANOVA) to examine the effect of nutrients and salinity pulse treatments on *Vallisneria* growth parameters, macroalgae, phytoplankton and selected environmental parameters. Raw data were square-root transformed when the residuals of the model did not have constant variance. All statistical analyses were conducted using JMP v. 5.0 [28]. Light attenuation and other environmental variables in the experimental tanks were similarly analysed. The response variables included the major components of macrophyte growth (above-ground, below-ground, total biomass, root : shoot ratio, areal productivity and LAI) and the three major groups of associated algal components (epiphyte biomass, water column chlorophyll *a* (representative of phytoplankton) and surface algal mat biomass). We also performed statistical analyses on environmental parameters to evaluate the consistency of each experimental tank's systems maintenance and to determine the response of environmental parameters to experimental conditions. Light attenuation coefficient measurements were included as a response variable because of the physical effect that macrophyte and algae have on water column solar irradiance. For statistical analyses, the environmental data were grouped into pre- and post-treatment monitoring periods.

## 4. Results

**Figure 1.**
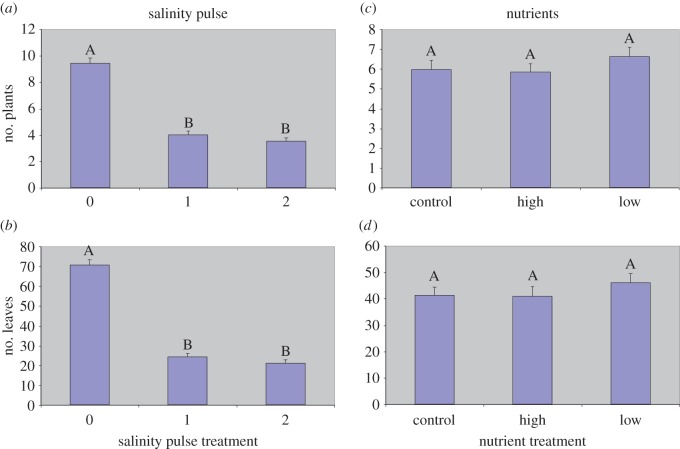
Mean number of plants (ramets) (*a*,*c*) and leaves (*b*,*d*) per pot measured at final harvest for each salinity pulse treatment (*a*,*b*) and each nutrient treatment (*c*,*d*). Levels not connected by same letter are significantly different at *p*=0.05.

We observed no significant treatment effects on root : shoot ratios, epiphytes and algal mat biomass (table 2). However, we did observe significant salinity effects on above-ground, below-ground, total biomass, aerial productivity and light attenuation. Salinity pulse treatments had a dramatic effect on all *Vallisneria* growth parameters and we observed a comparable response of plants to both the 1 and 2 salinity pulses in comparison with the control (table 2). A notable difference between 1- and 2-pulse treatments was in the amount of plant mortality. At final harvest, 22% of the 1-pulse treatment pots were void of vegetation while 36% of 2-pulse treatment pots were void of viable plant material (a difference of 14%). However, a more accurate indicator of salinity-induced plant mortality was observed in counts of individual ramets and leaves performed at final harvest (figure 1*a*,*b*).

**Table 2.** ANOVA for response variables to treatments of nutrients and salinity. *p*-values in bold indicate significance at *p*=0.05.

treatment	response variable	d.f.	*F* ratio	*p*-value
whole model	above-ground biomass	8	52.22	<**0.0001**
	below-ground biomass	8	14 617	<**0.0001**
	total biomass	8	51.5448	<**0.0001**
	root : shoot	8	1.1389	0.3446
	aerial productivity	8	5.15	<**0.0001**
	LAI	8	25.89	<**0.0001**
	epiphyte biomass	8	1.25	0.2998
	chlorophyll *a*	8	12.79	<**0.0001**
	algal mat biomass	8	1.44	0.2444
	light attenuation coefficient	8	2.9	**0.0052**
nutrients	above-ground biomass	2	1.46	0.2366
	below-ground biomass	2	1.32	0.2722
	total biomass	2	1.39	0.2523
	root : shoot	2	0.45	0.6398
	aerial productivity	2	0.15	0.8621
	LAI	2	3.25	**0.0480**
	epiphyte biomass	2	1.76	0.1861
	chlorophyll *a*	2	6.30	**0.0025**
	algal mat biomass	2	0.2308	0.7962
	light attenuation coefficient	2	1.48	0.2302
salinity	above-ground biomass	2	202.82	<**0.0001**
	below-ground biomass	2	128.99	<**0.0001**
	total biomass	2	200.07	<**0.0001**
	root : shoot	2	3.59	**0.0311**
	aerial productivity	2	20.22	<**0.0001**
	LAI	2	82.32	<**0.0001**
	epiphyte biomass	2	1.54	0.2293
	chlorophyll *a*	2	36.51	<**0.0001**
	algal mat biomass	2	4.8204	**0.0211**
	light attenuation coefficient	2	7.93	**0.0006**
salinity × nutrients	above-ground biomass	4	1.66	0.1647
	below-ground biomass	4	2.00	0.1000
	total biomass	4	1.77	0.1415
	root : shoot	4	0.23	0.9222
	aerial productivity	4	0.12	0.9758
	LAI	4	8.99	<**0.0001**
	epiphyte biomass	4	1.25	0.3058
	chlorophyll *a*	4	4.19	**0.0032**
	algal mat biomass	4	0.37	0.8291
	light attenuation coefficient	4	1.10	0.3554

The number of surviving plants and leaves were significantly greater in the control treatments than those of either salinity treatments and 1- and 2-pulse treatments did not differ. We determined a 59.7% ramet loss and 67.8% loss of leaves in the combined salinity treatments; a substantial increase from whole pot mortality estimates. However, when comparing plant and leaf numbers as a function of nutrient treatments, there were no significant differences among any of the treatments (figure 1*c*,*d*), indicative of the overwhelming effect salinity had on *Vallisneria* biomass and growth in comparison with the effect of nutrients.

**Figure 2.**
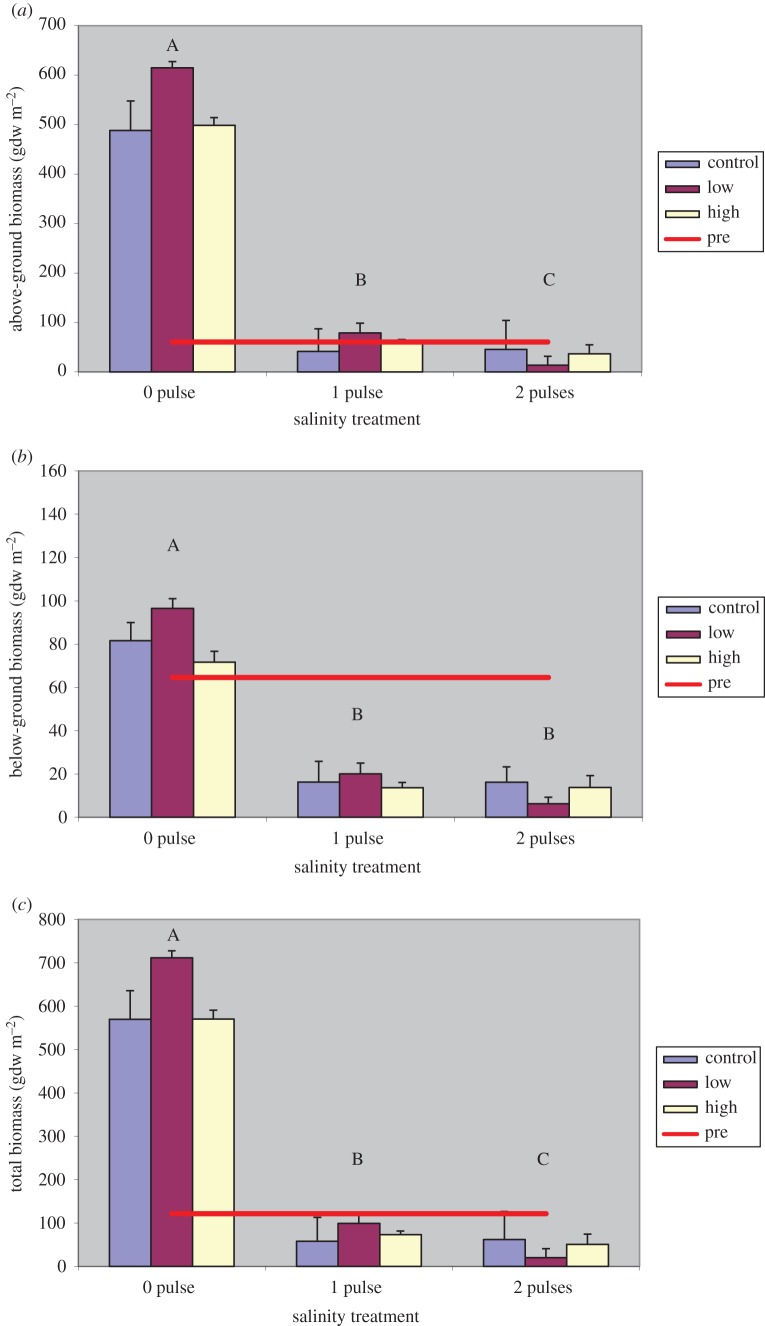
Mean *Vallisneria* above-ground (*a*), below-ground (*b*) and total biomass (*c*) at final harvest for each treatment. Bars represent different nutrient loading rates for each salinity treatment. The red line represents the pre-treatment mean. Levels not connected by same letter are significantly different at *p*=0.05.

Similar results are found in measures of above-ground, below-ground and total biomass (figure 2). Above-ground biomass was greatest in 0-pulse treatments and the 1-pulse treatment was significantly greater than the 2-pulse treatment (figure 2*a*). However, the difference in the mean above-ground biomass between 0- and 1-pulse treatments (−475 gdw m^−2^) was much greater than between 1- and 2-pulse treatments (−27.8 gdw m^−2^).

Below-ground biomass in the 0-pulse treatment was also much greater in the 1- and 2-pulse treatments (figure 2*b*), but the 1- and 2-pulse treatments were not significantly different, possibly indicating a higher salinity resistance in the below-ground component. Total biomass (figure 2*c*) was nearly identical to above-ground biomass with significantly greater biomass in 0-pulse treatments and with very little difference occurring between the salinity treatments.

**Figure 3.**
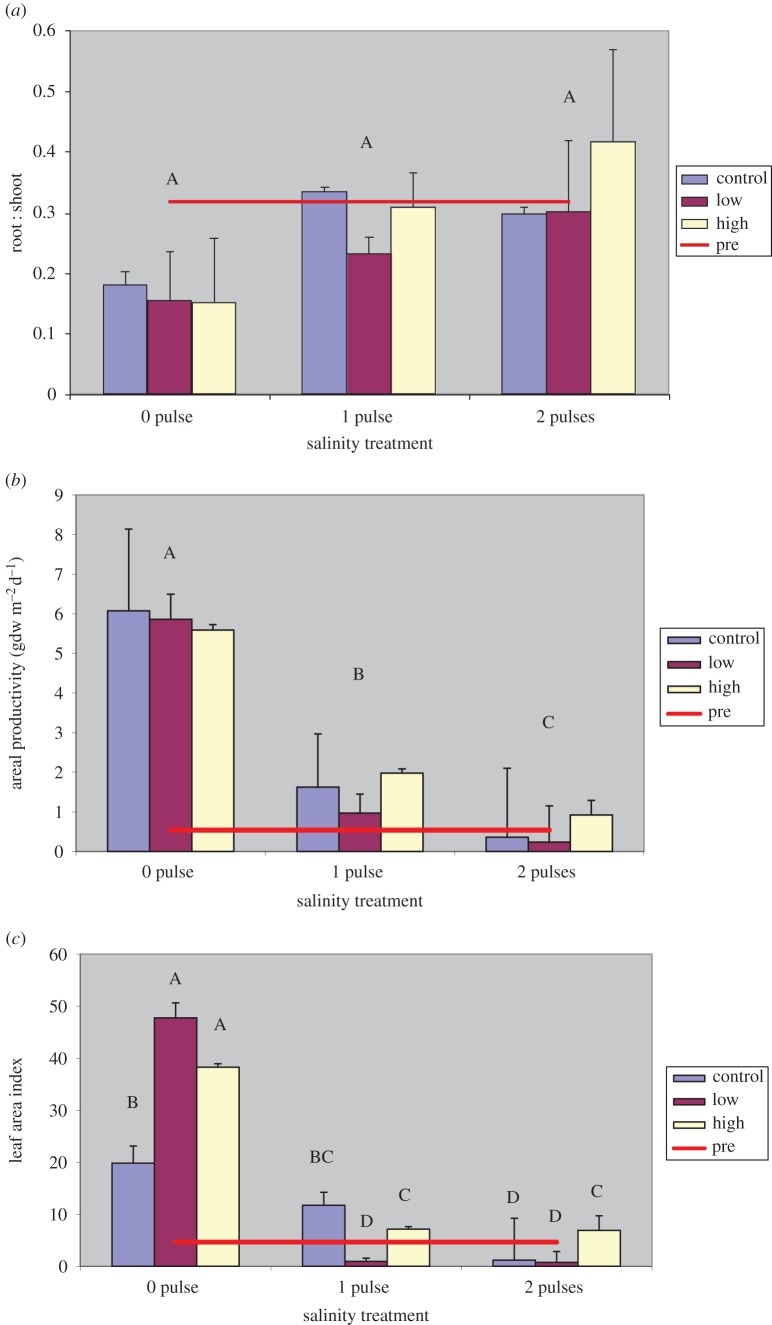
Mean *Vallisneria* root : shoot ratio (*a*), areal productivity (*b*) and LAI (*c*) at final harvest for each treatment. Bars represent different nutrient loading rates for each salinity treatment. The red line represents the pre-treatment mean. Levels not connected by same letter are significantly different at *p*=0.05.

Root : shoot ratios did not differ among any of the treatments (figure 3*a*). Above- and below-ground growth responded similarly to treatments, but while not statistically significant, the mean root : shoot ratios were slightly higher with salinity pulses, possibly indicating a gradual shift in biomass to roots with salinity exposure.

At final harvest, the 0-pulse treatments had significantly ( *p*<0.0001) higher areal productivity (figure 3*b*) than both pulse treatments, and the 1-pulse treatments had significantly higher areal productivity than the 2-pulse treatments. As a measure of the rate of new growth, the higher areal productivity in 1-pulse treatments in comparison with 2-pulse treatments may be indicative of recovery from the initial salinity pulse treatment.

The LAI (figure 3*c*) was the only macrophyte measurement to show a significant salinity by nutrient interaction ( *p*≤0.0001). At final harvest, the highest LAI were in the 0-pulse treatments, and both high- and low-nutrient regimes had significantly greater LAI than the controls. The LAI of plants exposed to 1-pulse, low-nutrient treatments, 2-pulse control and low-nutrient treatments were significantly lower than that of all other treatments. Under all nutrient regimes, 0-pulse treatments had greater LAI than the 1- and 2-pulse treatments, except in the control, where the 0- and 1-pulse treatments had greater LAI than the 2-pulse treatments.

**Figure 4.**
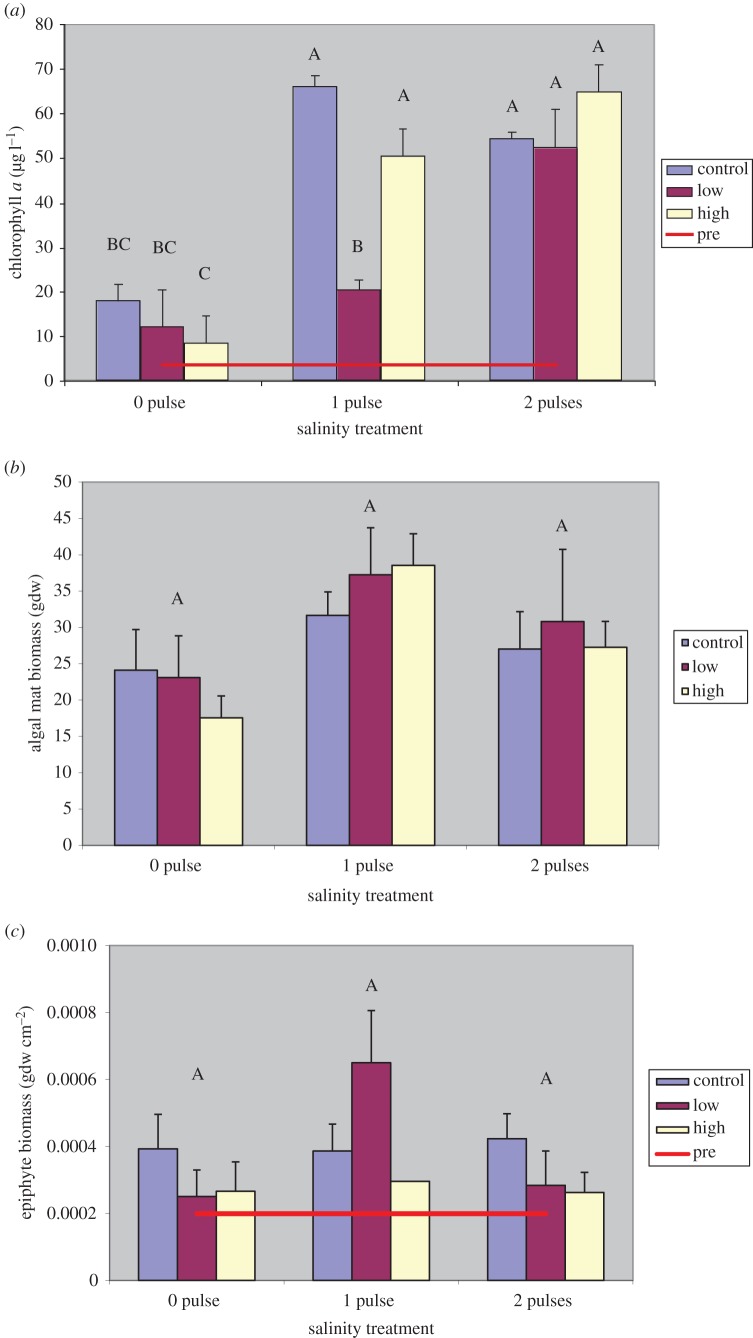
Mean water column chlorophyll *a* concentrations (*a*), surface algal mat (filamentous macroalgae) cover (*b*) and epiphyte biomass at final harvest (*c*) for each treatment. Bars represent different nutrient loading rates for each salinity treatment. The red line represents the pre-treament mean. Levels not connected by same letter are significantly different at *p*=0.05.

Chlorophyll *a* concentrations were variable among treatments throughout the experiment. Chlorophyll *a* concentrations had a significant salinity by nutrient interaction (table 2 and figure 4*a*) with all 2- and 1-pulse low-nutrient and 1-pulse high-nutrient treatments having similar and highest concentrations. Treatments of 1 pulse at control nutrient levels had similar concentrations to 0-pulse control and low nutrients, which did not differ significantly from 0-pulse high-nutrient treatments. Initially, we also observed widely varying degrees of surface algal mat coverage, but by final harvest, there was no difference regardless of treatment (figure 4*b*). As with surface macroalgae, we observed very little difference in epiphytes across treatments ( *p*=0.2998; figure 4*c*). At final harvest, the epiphyte loads were quite low, possibly as a result of the low light conditions brought about by heavy surface algae cover.

## 5. Discussion

Macrophyte response to nutrient enrichment tended to be subtle and complex, whereas the salinity response was much more direct and immediate. In fact, the effect of a single 18 ppt salinity pulse on *Vallisneria* from the LSJR reduced growth and survivorship substantially even though the exposure to the maximum salinity during the pulse was only for a period of 2 days. In a similar experiment on plants extracted from the LSJR, Jacoby [5] reported that after an exposure period of only 3–5 days to 18 ppt, *Vallisneria* plants exhibited significant blade and ramet loss, but salinity of up to 8.8 ppt produced no consistent decrease in viability over periods of exposure varying from 3 to 128 days. Our observations were that the difference in magnitude between 1 and 2 salinity pulses was relatively small in comparison to the difference between those plants exposed to salinity pulses and the 0-pulse treatments. The similar reduction in ramets and leaves at 59.7% and 67.8%, respectively, indicates that there was virtually no difference between pots that received a 1-pulse treatment of 18 ppt and pots that received the 2-pulse treatments of 18 and 12 ppt. This was also supported by the fact that 1- and 2-pulse treatments differed very little on almost every measure of macrophyte biomass and growth in comparison to 0-pulse treatments.

The lack of differences we observed in ramet and blade counts between the 1- and 2-pulse treatments can perhaps be explained by the possibility that *Vallisneria* was able to better tolerate the second pulse of 12 ppt than the first pulse of 18 ppt and, therefore, resulted in no additional plant mortality. But it is also possible that genetic variation exists within the population where plants that were less tolerant to salinity were lost in the first pulse and the remaining more salinity-tolerant plants were able to survive the second pulse [29]. A third possibility could be related to edaphic factors in which differences in substrate properties could allow some plants to store up greater nutritional reserves and improve salinity tolerance [4,30]. These possibilities remain speculative within the scope of this study; however, there apparently are factors involved, whether biotic or abiotic, that result in varying ability of individual plants within a population to survive salinity pulses. Perhaps what is most important for the purposes of management is that our study indicates that a portion of the population should survive, making recovery possible.

In above- and below-ground biomass, areal productivity and LAI, we observed greater means in the 1-pulse treatments than 2-pulse treatments. Total biomass of the combined 1-pulse treatments was 77.0 gdw m^−2^, which was 1.6 times greater than the 48.1 gdw m^−2^ in the 2-pulse treatments. Areal productivity was 3.0 times greater and LAI was 2.2 times greater in 1-pulse treatments. Although these means are much smaller than in 0-pulse treatments, they do nevertheless indicate that the 1-pulse treatment plants were recovering from the salinity exposure. Areal productivity, as a determinant of new growth, is perhaps the most indicative of recovery. The fact that a 3 times greater rate of growth in 1-pulse treatment plants compared with 2-pulse treatment plants at final harvest suggests that 1-pulse treated plants were increasing their rate of biomass assimilation. By final harvest, the rate of areal productivity in plants exposed to 1-pulse treatments was 1.52 gdw m^−2^ d^−1^, while the 2-pulse treatment plants were producing at a rate of only 0.5 gdw m^−2^ d^−1^.

**Figure 5.**
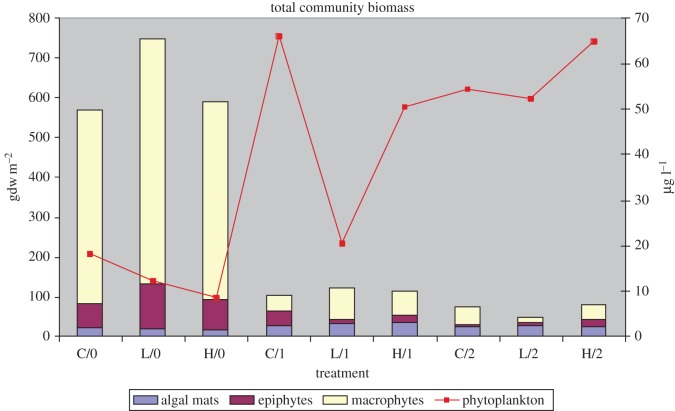
Summary of total community biomass at final harvest. Macrophytes, epiphytes and surface algal mats are represented on the primary *y*-axis expressed as gdw m^−2^. Phytoplankton is represented on the secondary *y*-axis as μg l^−1^ chlorophyll *a* concentration. Treatments are indicated on the *x*-axis; the symbols include the nutrient treatment (C, control; L, low; H, high) followed by the salinity pulse treatment (0, no pulse; 1, 1 pulse at 18 ppt; 2, 2 salinity pulses at 18 and 12 ppt). For example, L/1 is the treatment with the low-nutrient loading rate and 1 salinity pulse.

In a comparison of all of the vegetative components combined, it is apparent that salinity pulsing has a greater effect on the growth of the plants than the effect of variable nutrient loading rates as a treatment (figure 5). A linear regression of salinity pulse treatments against total biomass resulted in a correlation coefficient (*r*^2^) of 0.79, whereas nutrient treatments yielded a coefficient of 0.007. This observation should by no means de-emphasize the importance of nutrient loading on the long-term health of *Vallisneria* populations, but more obviate the difference in the immediate impact of salinity exposure as compared with nutrient treatments. In fact, as in our previous studies, we found that salinity had a direct and immediate impact on *Vallisneria* growth [31], but variable nutrient loading resulted in subtle changes to the macrophyte community and therefore full impact of nutrients may not be realized for several growing seasons [32]. These previous studies also showed that increased nutrients may increase algae biomass, which in turn competes with the macrophytes for light and nutrients.

Because of these community dynamics, it is also important to consider the effect salinity has on algae and how nutrients may influence these community interactions [33]. For example, in figure 5, it is apparent that macrophytes dominate the community in 0-pulse treatments. After 1-pulse treatments, there was a dramatic reduction in macrophyte biomass while phytoplankton was on the increase. However, in the 1-pulse low-nutrient treatment, phytoplankton biomass remained nearer to the levels of the 0-pulse treatments, indicating that lower nutrients may limit phytoplankton growth, whereas at control and high nutrients both 1- and 2-pulse treatments heavily favoured phytoplankton growth. The 2-pulse low-nutrient treatment did not result in the same response, but among the 1-pulse treatments the low-nutrient treatment had the highest combined macrophyte and macroalgae biomass, whereas in 2-pulse treatments the low-nutrient treatment had the lowest total biomass. The surplus nutrients as a result of lower combined macrophyte and macroalgae biomass may have been enough to maintain a higher level of phytoplankton growth by offsetting the lower nutrient loading.

In a previous study of constant salinity exposure [31], we found a very strong relationship between salinity and phytoplankton that seemed to result from a lack of competition by macrophytes and macroalgae. Salinity exposure greatly limited the growth of these other components and phytoplankton thrived, perhaps taking advantage of the lack of competition for nutrient resources. However, under control conditions (1 ppt salinity) and when salinity was lowered back down to 1 ppt from the 18 ppt salinity treatments, we observed a shift from phytoplankton dominance to macrophytes and macroalgae. In the current analysis, macrophytes tended to dominate the system under control conditions but we observed an increase in the prevalence of algal components with increased nutrients. The result, while not significant, was nevertheless a higher mean macrophyte biomass in low-nutrient loading treatments, suggesting that low nutrients could be the preferable condition to result in the dominance of macrophytes in the community. We observed a significant shift in community dominance to phytoplankton with a single salinity pulse (18 ppt) except in low-nutrient treatments where macrophytes prevailed. However, two salinity pulses in a growing season favoured algal dominance under all nutrient regimes as salinity had an immediate and overwhelming effect on macrophyte growth.

Doering *et al.* [20] found that the degree of *Vallisneria* mortality at exposure to 18 ppt was determined to be proportional to the duration of exposure. However, the mortality rates were much less than what we observed in the present analysis. They concluded by regression analysis that exposure of *Vallisneria* to 18 ppt salinity for 31 days would result in only 50% loss of shoots with 10% remaining after 95 days. They also determined that plants exposed to 18 ppt for 30 days could achieve a 50% recovery of lost blades and shoots during the following 30 days and plants exposed to 18 ppt for 15 days could fully recover. Although our final harvest biomass of plants exposed to a single 2-day pulse of 18 ppt salinity was only about 14% of what the 0-pulse treatments were, we did observe signs of recovery. The rates were much less than the estimates by Doering *et al.* [20] for *Vallisneria*. Frazer *et al.* [34] also found a reduction in all growth measures of *Vallisneria* exposed to 15 ppt salinity regardless of the duration of exposure. However, they also determined that all *Vallisneria* plants survived 1- and 2-day exposures to 15 ppt, whereas 25–100% of these plants died following 7-day exposure to 15 ppt. These findings suggest that while duration of exposure is important the species may tolerate storm-induced tidal surges and other short-term variations in salinity.

Salinity tolerance limits of *Vallisneria* vary in the literature. Under constant salinity conditions, cessation of growth has been reported to occur at 8.4 ppt [35,36], 6.66 ppt [37] and 15 ppt [16]. Twilley & Barko [14] found no effect of salinity at 12 ppt, and in other field analyses death occurred at salinities of 13.3 ppt [37] and in some cases at salinities higher than 15 ppt [15]. Differences between studies may be due to differences in methodology, methods of exposure or to real differences in salinity tolerance between populations [14,20]. Some of the main differences between our study and that of Doering *et al.* [20] were that they used indoor mesocosms lighted by metal halide lights, whereas our experimental set-up was located in a greenhouse with natural lighting. Doering *et al.* [20] used natural seawater from the Atlantic Ocean, while we used artificial sea salts. However, these differences alone were not likely to be responsible for such broad differences in salinity tolerance, since both experiments had experimental controls in place. Another key difference between the studies was that Doering *et al.* [20] actively removed macroalgae, and because they replaced the volume of their experimental tanks three times daily, phytoplankton development was held to a minimum. This experimental methodology effectively removed algal competition as a factor in the growth of the macrophytes, which certainly could be an important consideration in assessing community dynamics. However, in our study this may not have been critical with respect to salinity impact, because at the time of the first pulse treatment the tanks lacked any significant macro- and/or microalgae development. We did not see major algal development until the midpoint of the experiment, and the effect of salinity on *Vallisneria* was immediate, superseding any adverse effects of algae.

The most likely explanation for the variations in salinity tolerance may be that there are real differences in salinity response by populations of *Vallisneria*. These populations may be developing differences in salinity tolerance over time based on the frequency and duration of salinity exposure. For example, the site where Doering *et al.* [20] collected plants had peak mean salinities at 13–14 ppt, whereas the site where we collected plants was considered primarily freshwater with occasional oligohaline (less than or equal to 3 ppt) spikes. We observed evidence for individual variation in salinity tolerance within the population, in that even though some plants did not thrive, others did and recovered quite well once treatments were removed. However, we observed no plants that seemed to thrive under the experimental conditions, and our previous study showed that prolonged exposure to 18 ppt salinity would cause complete mortality in a growing season [31]. The fact that the duration of our exposures was much less than the Doering *et al.* [20] study further suggests that population differences may exist beyond simple individual plant differences in salinity tolerance. If in fact these population differences are real, then it would reinforce the idea that generalization about salinity tolerance of *Vallisneria* cannot be made and individual populations of *Vallisneria* should be assessed when making management decisions.

When considering the effects of nutrients and salinity on communities of *Vallisneria*, it is quite clear that the impact of salinity on *Vallisneria* is more immediate and obvious than that of nutrient treatments. Also, recovery from salinity impacts begin immediately after the salinity is reduced making it relatively easy to assess the damage. Unless major hydrologic changes have been made to an estuarine system, salinity is largely a function of natural forces, such as climate variations (e.g. drought) and storm events (e.g. storm surges) that, for the most part, cannot be controlled. The effects of such events may be catastrophic, but they are also event-driven, usually short-term, and typically limited to an upstream point where freshwater flow is able to offset the salinity intrusion. Recovery can begin as soon as the event, which may last for a few days or a full growing season, is over. Therefore, even though the effects of salinity are immediate and often locally catastrophic to populations of *Vallisneria*, the event is often short-lived and the site returns relatively quickly to recoverable or restorable conditions.

The effects of nutrients, on the other hand, seem to be much more complicated and the impacts may take much longer to manifest themselves. While salinity has a direct toxic effect, nutrients are much more insidious causing community changes, which invoke competitive forces that ultimately result in negative feedback to macrophytes. Because of the complexity of mechanisms involved in nutrient enrichment, recovery in large ecosystems affected by eutrophication may take several years and quite possibly decades to recover, requiring complicated and costly management approaches that affect very broad areas and multiple interests.

## Supplementary Material

The data supplementary material contains all of our raw sample data for each of the parameters measured. The file is named “SJR03 Pulse-Nut RawData-Boustany”

## Data accessibility

The datasets supporting this article have been uploaded as part of the electronic supplementary material. Additional information repository for the project material is located at the US Geological Survey, National Wetlands Research Center in Lafayette, LA, USA.
